# Recognizing Early Deterioration in Elderly Care Home Setting - a Snapshot

**DOI:** 10.1192/bjo.2022.160

**Published:** 2022-06-20

**Authors:** Ioana Varvari, Pratibha Nirodi

**Affiliations:** 1 Tees, Esk and Wear Valley NHS Foundation Trust, York, United Kingdom; 2 Tees, Esk and Wear Valley NHS Foundation Trust, Harrogate, United Kingdom; 3 Health Education England, York and Humber, United Kingdom

## Abstract

**Aims:**

Our aim is to measure the baseline physical and mental health early deterioration recognition of carers in the care home setting in Harrogate, North Yorkshire. This is part of a larger undergoing quality improvement project that looks at improving elderly care in care homes in the region by implementing a training package.

**Methods:**

The approach was to contact local authorities, in this case, the NHS clinical commissioning group North Yorkshire to identify a struggling care home. We then engaged the care home and designated a leader to coordinate the project. We collected common themes by using focus groups with both carers and our professionals which led to the creation of a 16-item questionnaire covering deterioration literacy. Finally, we electronically and anonymously surveyed the carers (December 2021) and analysed the data via Google Forms.

**Results:**

We had 22 responses out of 30 possible. As an overview, 100% felt confident in recognizing deterioration, however, 31.8% don't feel confident in managing deterioration. 90.9% need tools to aid recognition, from which 45.5% find tools confusing. Only 50% feel confident to appropriately escalate the incident, from which 36.4% did not know when or to whom to escalate and 13.6% were not sure if escalation was needed but will refer to secondary care regardless. 27.3% think their escalation process needs improvement. When it comes to deterioration themes, 4.6% don't feel confident in identifying confusion, 13.6% feel their knowledge on confusion could be improved and 9.1% don't know how to identify, manage, or escalate confusion. 22.7% don't feel confident in identifying mobility decline and 9.1% don't know how to manage this accordingly. 9.1% feel like their knowledge of skin changes needs improvement. 22.7% feel that their confidence in identifying toilet habits could be improved and 4.5% don't know how to manage or escalate these changes. In terms of carers’ mental health, 50% and 13.6% have mild and moderate anxiety, respectively.

**Conclusion:**

Deterioration recognition in the elderly is currently a hot topic. Recent studies highlight the need to improve deterioration management to minimize inappropriate referrals and admissions and unnecessary infection exposure of a vulnerable elderly individual. Our results show that besides improving the theoretical knowledge we also must think about a clear escalation process, an easy-to-read deterioration tool, and managing carers' anxiety as part of the training package.

